# Incidence and Risk Factors for Antiplatelet Therapy–Related Bleeding Complications Among Elderly Patients After Coronary Stenting: A Multicenter Retrospective Observation

**DOI:** 10.3389/fphar.2021.661619

**Published:** 2021-07-30

**Authors:** Yanxia Qian, Bing Xu, Xiaodong Qian, Lu Cao, Yujia Cheng, Xinjian Liu, Song Bai, Zhijun Han, Junhong Wang

**Affiliations:** ^1^ Department of Cardiology, The First Affiliated Hospital With Nanjing Medical University, Nanjing, China; ^2^ Department of Cardiology, Northern Jiangsu Province Hospital and Clinical Medical College, Yangzhou University, Yangzhou, China; ^3^ Department of Cardiology, The First Affiliated Hospital of Soochow University, Suzhou, China; ^4^ Department of Pathogenic Microorganism, Nanjing Medical University, Nanjing, China; ^5^ Key Laboratory of Antibody Technique of National Health Commission of China, Department of Pathogen Biology, Nanjing Medical University, Nanjing, China; ^6^ Department of Cardiology, Xuyi People’s Hospital, Huai’an, China; ^7^ Department of Laboratory Medicine, The Affiliated Wuxi No.2 People’s Hospital of Nanjing Medical University, Wuxi, China

**Keywords:** bleeding risk, dual antiplatelet therapy, elderly patients, PRECISE-DAPT score, risk factors

## Abstract

**Purpose:** To determine the incidence and risk factors of bleeding events as well as assess the performance of the PRECISE-DAPT score in elderly patients (≥75 years) who underwent percutaneous coronary intervention (PCI) and one-year dual antiplatelet therapy (DAPT).

**Methods:** A total of 940 patients (≥75 years) who received PCI and one-year DAPT were retrospectively enrolled into the study. The multivariable logistic regression analysis was conducted to identify risk factors of antiplatelet-related bleeding complications. The receiver operating characteristic (ROC) curve analysis and the Delong test were performed to obtain the optimized PRECISE-DAPT score.

**Results:** It was observed that 89 (9.47%) patients suffered bleeding complications, while 37 (3.94%) of them had the Bleeding Academic Research Consortium (BARC, type ≥2) bleeding events. We stratified the PRECISE-DAPT score in tertiles (T1: ≤23; T2:24 to 32; T3: ≥33) and found that BARC ≥ 2 type bleeding occurred more frequently in T3 than in T1 and T2 (8.25 vs. 1.46% vs. 2.40%, *p* <0.05). The ROC curve analysis revealed that the PRECISE-DAPT score cutoff for BARC ≥2 type bleeding prediction was 33. In comparison with the current recommended cutoff score of 25 (AUC: 0.608, based on ROC analysis), the Delong test indicated significantly improved ability for predicting BARC ≥ 2 type bleeding events using the proposed cutoff value of 33, AUC of 0.676 (*p* = 0.03), and Brier Score of 0.04. The multivariable logistic regression analysis demonstrated that the PRECISE-DAPT score ≥ 33 [OR: 3.772; 95% CI (1.229, 11.578); *p* = 0.02] was associated with BARC ≥ 2 type bleeding event, along with a history of hemorrhagic stroke [OR: 6.806; 95% CI (1.465, 31.613); *p* = 0.014], peptic ulcer [OR: 3.871; 95% CI (1.378, 10.871); *p* = 0.01], and/or myocardial infarction [MI, OR: 3.081; 95% CI (1.140, 8.326); *p* = 0.027].

**Conclusion:** A higher PRECISE-DAPT score of 33 might be a more reasonable cutoff value for predicting BARC ≥2 type bleeding risk in CAD patients (≥75 years). In addition, the history of hemorrhagic stroke, peptic ulcer, and myocardial infarction were identified as the risk factors of BARC ≥2 type bleeding events.

## Introduction

Age is a risk factor for coronary artery diseases (CADs) (Shanmugam et al., 2015). Thus, an increasing number of the elderly population (aged ≥75 years) usually develop CAD, wherein percutaneous coronary intervention (PCI) is considered as the definitive treatment option. Dual antiplatelet therapy (DAPT), consisting of aspirin with a P2Y12 receptor inhibitor (mainly clopidogrel and ticagrelor in participating centers), is an essential treatment regimen in patients undergoing PCI with decreased risks of ischemic complications but increased risks of bleeding, wherein the condition can sometimes be fatal and culminate in adverse clinical events (Wang et al., 2011; Levine et al., 2016; Tammam et al., 2017; Palmerini et al., 2017a; Xu et al., 2018). Elderly patients are a special group in CAD studies since they present with extensive atherosclerosis, complex lesions, comorbidities, and increased incidences of both thrombosis and bleeding (Wang et al., 2011; Shanmugam et al., 2015; Arahata and Asakura, 2018). Nevertheless, an existing evidence showed that the elderly remained as an underrepresented group in clinical trials (Dodd et al., 2011). Bleeding risk is regarded as an important limiting factor that affects patient’s decision for the antithrombotic strategies (Almendro-Delia et al., 2017), and thus, existing guidelines recommend careful consideration for complications of bleeding risk during DAPT (Valgimigli et al., 2017; Kedhi et al., 2018; Mazlan-Kepli et al., 2019). Besides, a careful assessment of bleeding risk may change the prognosis of the patients. Several bleeding risk scores, namely, CRUSADE, ACUITY, and PRECISE-DAPT scores, have been proposed *albeit* having a lower representation of the elderly patients. Notwithstanding, the PRECISE-DAPT score is recommended (by the European Society of Cardiology guidelines) as a guide for the length of DAPT (Subherwal et al., 2009; Mehran et al., 2010; Ariza-Solé et al., 2014; Faustino et al., 2014; Valgimigli et al., 2017; Choi et al., 2018). The PRECISE-DAPT score is a simple tool that was developed with five items (age, creatinine clearance, hemoglobin, white blood cell count, and previous spontaneous bleeding). Importantly, it can be applied in daily clinical practice to assess bleeding risks during the initiation of treatment (Costa et al., 2017). Current guidelines suggest a relative conservative antiplatelet approach in patients with the PRECISE-DAPT score above the recommended 25 cutoff value (Valgimigli et al., 2017). However, only few studies have assessed the predictive ability of the PRECISE-DAPT score in CAD patients (≥75 years). In this study, we investigated the clinical factors of the Bleeding Academic Research Consortium (BARC, type ≥2) bleeding events in elderly patients (≥75 years) who received coronary stenting and one-year DAPT. Also, the performance of the PRECISE-DAPT score to predict bleeding complications in patients (≥75 years) was evaluated.

Of note, this article has been presented in accordance with the STROBE reporting checklist.

## Methods

This was a multicenter, retrospective study conducted among CAD patients (≥75 years) who received DAPT after PCI, with implantation of drug-eluting stents (DESs) from three hospitals (viz., the first affiliated hospital with Nanjing Medical University, the first affiliated hospital of Soochow University, and affiliated hospital of Yangzhou University), regardless of being presented with acute coronary syndromes or chronic coronary syndromes (from September 2016 to June 2018). All the CAD patients who met the inclusion criteria were retrospectively selected from the medical electronic database at the three centers. The criteria were as follows: 1) age equal to or older than 75 years, 2) patients who received PCI treatment between September 2016 and June 2018, and 3) all the patients received one-year DAPT therapy, except for some of them changed to single antiplatelet therapy (SAPT) treatment due to bleeding complications. In addition, patients were excluded due to the following reasons: 1) contraindications to DAPT, namely, active bleeding, peptic ulcer, or allergic to one or more antiplatelet drugs; 2) bleeding complications that were not caused by antiplatelet therapy, such as trauma; 3) hematological disorders; 4) complications of serious diseases like malignant tumor with less than 1-year life expectancy; and 5) incomplete follow-up data due to loss of contact. Furthermore, patients with a history of PCI intervention or coronary artery bypass surgery within 1 year before enrollment were also excluded in order to rule out a possible increased bleeding risk associated with prolonged DAPT. The study protocol was conducted in accordance with the Declaration of Helsinki and was approved by the institutional ethics committee of each participating center (No. 2020-SR-472). Informed consent of the participants was waived in the study and approved by our ethical committee.

The clinical and laboratory variables at the time of index PCI for the analysis were collected retrospectively from the hospital medical electronic database. The clinical parameters included demographics, risk factors and previous histories, clinical presentations, laboratory examinations, angiographic and procedural characteristics, PRECISE-DAPT score, and discharge medication information. All patients who met the inclusion criteria were called (at least 1 year) after admission for the PCI in order to obtain the end point information. End point events referred to those bleeding complications that happened at any time between the starting of the DAPT regimen and the end of the 1-year follow-up time. Furthermore, the bleeding outcomes were further confirmed in some of the patients by checking their outpatient visit record or reviewing their related medical electronic record. The creatinine clearance rate (cCr) was calculated using Cockcroft–Gault equation. The PRECISE-DAPT score was calculated for all the patients through the web calculator (http://www.precisedaptscore.com/predapt/).

The clinical bleeding outcomes included complications caused by DAPT within 1 year after PCI in accordance with the BARC criteria (Supplementary Table S1) (Mehran et al., 2011). Bleeding events were subgrouped by BARC ≥2 type bleeding events (excluding BARC four type) and BARC <2 type bleeding events.

Continuous variables were presented as mean ± SD for normally distributed variables or median with IQR (interquartile range) (Q1–Q3) for non-normally distributed variables, while categorical variables were expressed as frequencies (percentages). Continuous variables were compared using the one-way ANOVA or Kruskal–Wallis H test, while the Pearson χ^2^ or Fisher exact test was used to compare categorical parameters. The incidence of bleeding events was calculated by dividing the number of patients who had bleeding events with the total number of patients in the subgroup. Since advanced age may push the PRECISE-DAPT score toward right, we stratified the PRECISE-DAPT score into tertiles to see if a higher score may relate to a higher incidence of bleeding events because the current recommended cutoff value of 25 might be too low for elderly patients in clinical practice. Furthermore, the optimal cutoff value of the PRECISE-DAPT score for the elderly patients was obtained through the receiver operating characteristic (ROC) curve analysis. In order to compare the predictive ability of bleeding events between the newly proposed PRECISE-DAPT cutoff score of 33 and the current recommended score of 25 in elderly patients, two cutoff values were, respectively, calculated for ROC curves and Brier scores. Meanwhile, the Delong test was used to determine the difference between two areas under the curves (AUCs) of ROC curves. Variables that were statistically significant in the univariate analysis were further analyzed in the multivariate logistic regression analysis. The univariate and multivariable binomial logistic regression analyses were performed to determine the clinical risk factors of bleeding events, and the results were expressed as the odds ratios (ORs) with corresponding 95% confidence intervals (CIs). All statistical analyses were performed using SPSS23.0 software or R language software, where appropriate. A two-sided *p*‐value of <0.05 was considered statistically significant.

## Results

### Baseline Characteristics

A total of 940 CAD patients (aged ≥75 years) who received PCI treatment were enrolled in this study. Their mean age was 79.54 ± 3.96 years, with 69.57% of the patients being male. Among them, 12.13% of the patients were 85 years or older. Meanwhile, 177 patients (18.83%) were diagnosed with stable angina (SA), 453 patients (48.19%) with unstable angina (UA), 119 patients (12.66%) with non–ST-segment elevation myocardial infarction (NSTEMI), and 191 patients (20.32%) with ST-segment elevation myocardial infarction (STEMI). In terms of the DAPT regimen, 721 patients (76.70%) used aspirin with clopidogrel, while 219 patients (23.30%) used aspirin with ticagrelor after discharge. Within 1 year after PCI, 89 patients (9.47%) suffered from bleeding complications, namely, 37 (3.94%) with BARC ≥2 (excluding BARC 4) bleeding events and 52 (5.53%) with BARC <2. Notably, 46 patients switched to SAPT due to bleeding events, while five permanently stopped antiplatelet treatment owing to recurrent bleeding complications. The most common locations where bleeding occurred were digestive, intracranial, nasal and oral, or urological. Characteristics of the patients are shown in Table 1. Compared with the no bleeding group, patients with BARC (type ≥2) bleeding events had a significantly lower cCr (52.49 ± 20.01 vs. 63.91 ± 27.33 ml/min, *p* = 0.031) and a higher PRECISE-DAPT score (37.68 ± 12.83 vs. 29.26 ± 10.41, *p* < 0.001). More patients with BARC ≥2 type bleeding events showed a history of myocardial infarction (MI) (16.22 vs. 5.29%, *p* = 0.011), hemorrhagic stroke (8.11 vs. 0.71%, *p* = 0.005), and peptic ulcer (16.22 vs. 3.17%, *p* = 0.002) than the patients without bleeding events. In comparison with the BARC <2 type bleeding event group, patients in the BARC ≥2 type bleeding event group had a higher PRECISE-DAPT score (37.68 ± 12.83 vs. 28.98 ± 9.63, *p* < 0.001), while more patients exhibited a history of PCI (24.32 vs. 5.77%, *p* < 0.05) and peptic ulcer (16.22 vs. 0%, *p* = 0.004).

**TABLE 1:** Baseline characteristics of patients with or without bleeding events.

	No bleeding event group (n = 851)	BARC<2 bleeding event group (n = 52)	BARC≥2 bleeding event group (n = 37)	*p* value
Demographics	—	—	—	—
Age, y	79.48 ± 3.91	79.85 ± 4.60	80.46 ± 4.17	0.286
Male (%)	594 (69.80)	35 (67.31)	25 (67.57)	0.897
Previous history	—	—	—	—
Hypertension (%)	651 (76.50)	42 (80.77)	28 (75.68)	0.770
Diabetes mellitus (%)	234 (27.50)	15 (28.85)	13 (35.14)	0.590
Smoking history (%)	274 (32.20)	21 (40.38)	12 (32.43)	0.474
Family history (%)	21 (2.47)	1 (1.92)	1 (2.70)	0.858
Previous MI (%)	45 (5.29)	3 (5.77)	6 (16.22)	0.028
Previous PCI (%)	131 (15.39)	3 (5.77)	9 (24.32)	0.050
Previous CABG (%)	9 (1.06)	2 (3.85)	1 (2.70)	0.096
Ischemic stroke (%)	91 (10.69)	7 (13.46)	8 (21.62)	0.098
Hemorrhagic stroke (%)	6 (0.71)	2 (3.85)	3 (8.11)	0.002
Peptic ulcer (%)	27 (3.17)	0 (0)	6 (16.22)	0.001
Peripheral vascular diseases (%)	14 (1.65)	0 (0)	0 (0)	1.000
HF (%)	21 (2.47)	2 (3.85)	2 (5.41)	0.264
AF (%)	69 (8.11)	6 (11.54)	2 (5.41)	0.574
Clinical presentation	—	—	—	0.790
SAP (%)	165 (19.39)	7 (13.46)	5 (13.51)	—
UAP (%)	404 (47.47)	30 (57.69)	19 (51.35)	—
NSTEMI (%)	108 (12.69)	5 (9.62)	6 (16.22)	—
STEMI (%)	174 (20.45)	10 (19.23)	7 (18.92)	—
SBP, mmHg	134.05 ± 21.40	137.35 ± 26.59	134.14 ± 23.57	0.685
DBP, mmHg	75.42 ± 12.51	73.25 ± 11.92	73.24 ± 13.78	0.300
HR, bpm	75.03 ± 14.08	77.38 ± 18.75	73.19 ± 10.72	0.397
Laboratory examinations at admission				
Hgb, g/L	127.50 ± 17.44	128.83 ± 17.57	124.54 ± 16.95	0.507
PLT, *10^9/L	182.09 ± 60.98	185.63 ± 42.04	203.27 ± 70.99	0.193
WBC, *10^9/L	7.41 ± 4.40	7.79 ± 3.33	7.41 ± 2.32	0.826
TC, mmol/L	3.95 ± 1.00	3.95 ± 0.89	3.89 ± 1.01	0.927
TG, mmol/L	1.40 ± 0.73	1.37 ± 0.97	1.25 ± 0.41	0.099
LDL-C, mmol/L	2.32 ± 0.81	2.34 ± 0.65	2.29 ± 0.89	0.961
HDL-C, mmol/L	1.10 ± 0.29	1.08 ± 0.25	1.06 ± 0.25	0.770
cCr, ml/min/1.73m^2	63.91 ± 27.33	62.06 ± 23.31	52.49 ± 20.01	0.039
PT (s)	13.07 ± 2.48	12.96 ± 1.65	13.03 ± 1.65	0.943
Diseased vessels	—	—	—	0.498
1-Vessel (%)	580 (68.16)	32 (61.54)	23 (62.16)	—
2-Vessels (%)	153 (17.98)	10 (19.23)	10 (27.03)	—
3-Vessels (%)	118 (13.87)	10 (19.23)	4 (10.81)	—
Stent numbers	1.64 ± 0.96	1.76 ± 1.21	1.78 ± 0.98	0.500
PRECISE-DAPT score	29.26 ± 10.41	28.98 ± 9.63	37.68 ± 12.83	<0.001
DAPT regimen	—	—	—	0.748
Aspirin + clopidogrel (%)	650 (76.38)	41 (78.85)	30 (81.08)	—
Aspirin + ticagrelor (%)	201 (23.62)	11 (21.15)	7 (18.92)	—
TT (%)	33 (3.88)	6 (11.54)	1 (2.70)	0.043
Other medications				
ACEI or ARB (%)	312 (36.66)	17 (32.69)	19 (51.35)	0.155
*β*-blockers (%)	420 (49.35)	31 (59.62)	19 (51.35)	0.351
PPI (%)	367 (43.13)	27 (51.92)	23 (62.16)	0.039

Data are expressed as mean ± SD, medians (25th–75th percentiles), or number (percentage).

BARC, Bleeding Academic Research Consortium; MI, myocardial infarction; PCI, percutaneous coronary intervention; CABG, coronary artery bypass grafting; HF, heart failure; AF, atrial fibrillation; SAP, stable angina pectoris; UAP, unstable angina pectoris; NSTEMI, non–ST-segment elevation myocardial infarction; STEMI, ST-segment elevation myocardial infarction; SBP, systolic blood pressure; DBP, diastolic blood pressure; HR, heart rate; Hgb, hemoglobin; PLT, platelets; WBC, white blood count; TC, total cholesterol; TG, triglyceride; LDL-C, low-density lipoprotein cholesterol; HDL, high-density lipoprotein cholesterol; cCr, creatinine clearance rate; PT, prothrombin time; ACEI, angiotensin converting enzyme inhibitor; ARB, angiotensin receptor blocker; TT, triple therapy; PPI, proton pump inhibitor.

### Performance of the PRECISE-DAPT Score in Predicting the Bleeding Events in Elderly Patients

**FIGURE 1:**
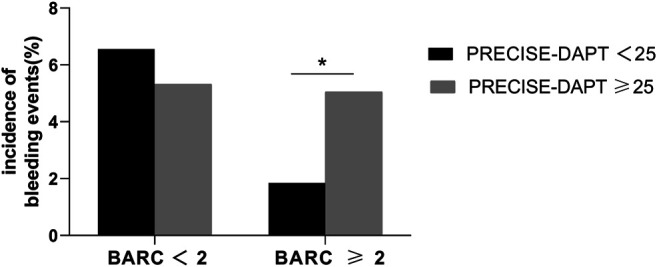
Incidence of bleeding events in patients with the PRECISE-DAPT score <25 or ≥25. The incidence of BARC ≥2 type bleeding events was higher in patients with the PRECISE-DAPT score ≥25 (5.05%) than in those with the PRECISE-DAPT score <25 (1.84%). * indicates *p* < 0.05. No significant difference was observed in BARC <2 bleeding events.

**FIGURE 2:**
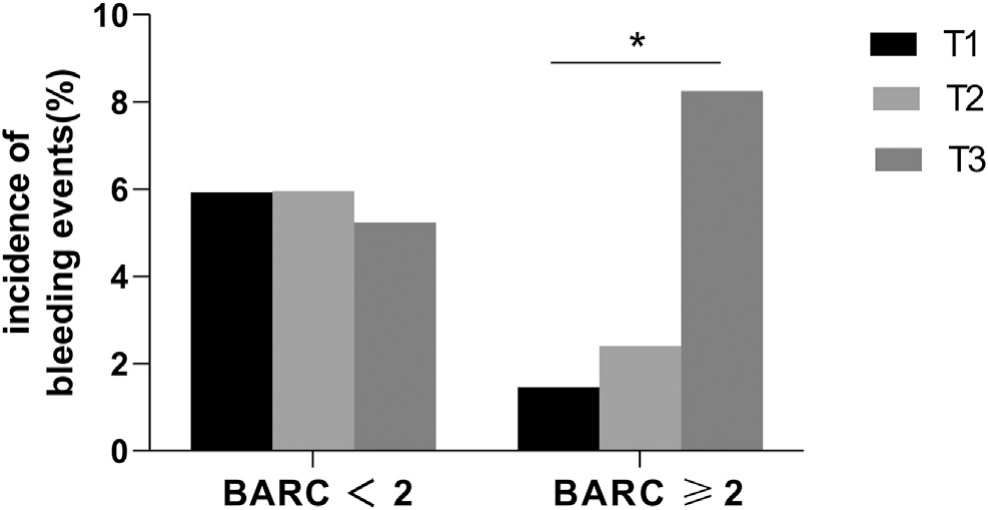
Incidence of bleeding events in low (T1), medium (T2), and high (T3) PRECISE-DAPT score groups. Low PRECISE-DAPT score: <the 33rd percentile (≤23), medium PRECISE-DAPT score: from the 33rd percentile to the 67th percentile (24–32), and high PRECISE-DAPT score: >the 67th percentile (≥33). The incidence of BARC ≥2 type bleeding events was significantly higher in the high PRECISE-DAPT score group (8.25%) than in the low (1.46%) and middle (2.40%) groups. * indicates *p* < 0.05. No significant difference was observed in BARC <2 bleeding events.

Patients were divided into low score (<25) and high score groups (≥25) according to the recommended PRECISE-DAPT score cutoff value of 25 (Figure 1). In the high-score group, the incidence of BARC ≥2 type bleeding events was significantly higher (5.05 vs. 1.84%, *p* = 0.016) than that in the low-score group. However, no significant difference was observed in BARC <2 type bleeding events between the two groups (5.32 vs. 6.56%, *p* = 0.442). We further stratified the PRECISE-DAPT score into low [<the 33rd percentile (≤23)], middle [the 33rd to 67th percentile (Schoenenberger et al., 2016; Sharma et al., 2017; Garay et al., 2018; Guerrero et al., 2018; Chen et al., 2019; Costa et al., 2019a; Pavasini et al., 2019; Shimizu et al., 2019; Martí et al., 2020)], and high [>the 67th percentile (≥33)] groups according to the level of the percentile of PRECISE-DAPT score (Figure 2). The incidence of BARC ≥2 type bleeding events was significantly higher in the high-score group (8.25%) than that in the low (1.46%) and middle (2.40%) groups, indicating that the higher the PRECISE-DAPT score, the more likely BARC ≥2 type bleeding events may occur. On the contrary, no significant difference was observed during the comparison of BARC <2 type bleeding events within the three groups (5.93 vs. 5.96% vs. 5.24%, *p* = 0.934).

**FIGURE 3:**
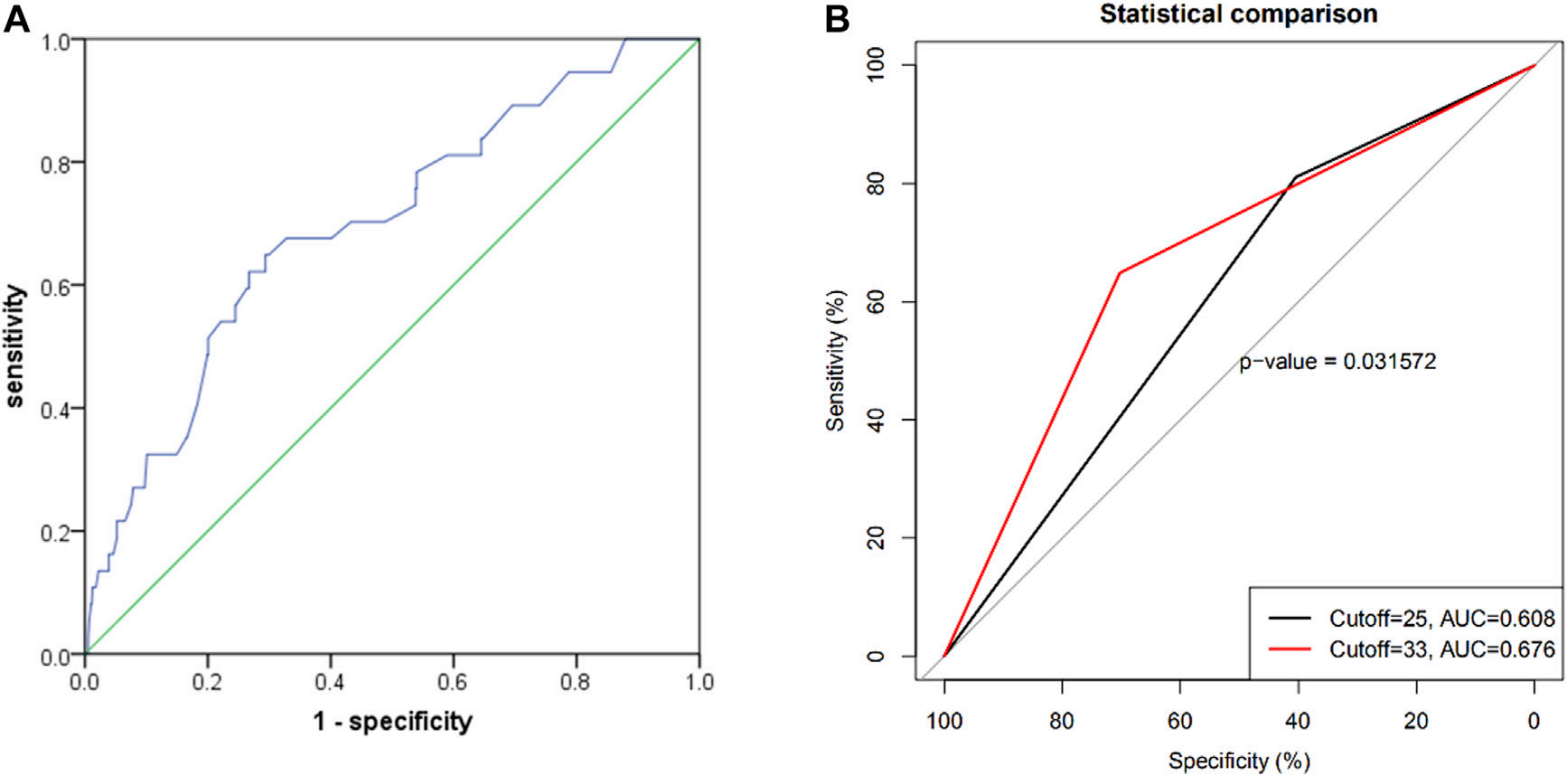
**(A)** Receiver operating characteristic curve for the prediction of BARC ≥2 type bleeding events by PRECISE-DAPT risk score systems in elderly patients with 1-year DAPT after PCI [AUC: 0.697; 95% CI (0.609, 0.784); *p* < 0.001]; **(B)** the comparison between the ROC curves of the PRECISE-DAPT score of 25 and the newly proposed cutoff score of 33 for prediction on the 1-year BARC ≥2 type bleeding events (*p* = 0.03).

The ROC curve of the PRECISE-DAPT score for BARC ≥2 type bleeding prediction is shown in Figure 3A, with an area under the curve (AUC) of 0.697 [95% CI (0.609, 0.784); *p* < 0.001]. The cutoff value obtained for the PRECISE-DAPT score *via* the ROC curve was 33, which was similar to the 67th percentile of the same score. When compared to the ROC of the current recommended cutoff value of 25 (AUC: 0.608), the cutoff value of 33 revealed an AUC of 0.676 (*p* = 0.03 with Delong test) (Figure 3B) and a Brier score of 0.04, indicating a significant improvement in the ability of newly proposed 33 (cutoff value) to predict BARC ≥2 type bleeding events in elderly patients.

### Risk Factors of BARC ≥2 Type Bleeding Events in Elderly Patients With One-Year DAPT After PCI

The univariate logistic regression analysis showed that the PRECISE-DAPT score ≥33, history of MI, ischemic stroke, hemorrhagic stroke and peptic ulcer, combined usage of PPI, and cCr were independent risk factors for BARC ≥2 type bleeding events. Multivariable logistic regression analysis further demonstrated that the PRECISE-DAPT score ≥33 [OR: 3.772; 95% CI (1.229, 11.578); *p* = 0.02] remained a significant risk factor for BARC ≥2 type bleeding events, together with the history of hemorrhagic stroke [OR: 6.806; 95% CI (1.465, 31.613); *p* = 0.014], peptic ulcer [OR: 3.871; 95% CI (1.378, 10.871); *p* = 0.01], or previous MI [OR: 3.081; 95% CI (1.140, 8.326); *p* = 0.027] (Table 2).

**TABLE 2:** Logistic regression analysis for bleeding events.

	Univariable analysis OR (95% CI)			Multivariable analysis OR (95% CI)		
	OR	95% CI	*p* value	OR	95% CI	*p* value
Previous hemorrhagic stroke	12.426	(2.981, 51.806)	0.001	6.806	(1.465, 31.613)	0.014
Previous peptic ulcer	5.907	(2.274, 15.342)	<0.001	3.871	(1.378, 10.871)	0.010
PRECISE-DAPT score	—	—	—	—	—	—
≤23	1 (ref)	—	—	1 (ref)	—	—
24–32	1.661	(0.506, 5.455)	0.403	—	—	—
≥33	6.024	(2.061, 17.609)	0.001	3.772	(1.229, 11.578)	0.020
Previous MI	3.467	(1.376, 8.736)	0.008	3.081	(1.140, 8.326)	0.027
Previous ischemic stroke	2.304	(1.023, 5.191)	0.044	—	—	—
Combination usage of PPI	2.167	(1.1, 4.268)	0.025	—	—	—
cCr, ml/min/1.73m^2	0.981	(0.966, 0.996)	0.012	—	—	—

OR, odds ratio; CI, confidence interval; MI, myocardial infarction; cCr, creatinine clearance rate; PPI, proton pump inhibitor.

## Discussion

In this retrospective study of patients (aged ≥75 years) who were treated with DAPT following PCI, two main findings were revealed (Shanmugam et al., 2015): the PRECISE-DAPT is an effective score system which can predict one-year bleeding events in elderly patients over 75 years. However, a higher score of 33, rather than the recommended 25, might be a more reasonable cutoff value for the PRECSE-DAPT score to diagnose BARC ≥2 type bleeding complications in elderly patients (Wang et al., 2011). The history of hemorrhagic stroke, peptic ulcer, and MI were the predominant risk factors associated with BARC ≥2 type bleeding complications in CAD patients (aged ≥75 years) who received PCI and one-year DAPT.

DAPT-related bleeding which led to higher mortality, lower life quality, and higher medical service charges has been identified as a common complication after PCI (Khandelwal et al., 2012; Amin et al., 2013). A higher incidence of bleeding complications has been found to be common in acute coronary syndrome patients (over 75 years) than in their younger counterparts (Alonso Salinas et al., 2018). Thus, antiplatelet therapy–associated bleeding risk increases with age (Li et al., 2017). To buttress this point, Oxford Vascular Study Group recently analyzed 3,166 patients who received secondary prevention (mostly with aspirin). The authors observed that the general bleeding risk in patients (≥75 years) increased by a 3.1 fold with the risk of fatal bleeding also increasing by 5.5 fold (Arahata and Asakura, 2018). Recently, an increased bleeding risk in older adults has been ascribed to more cardiovascular risk factors, complex anatomical structures, additional comorbidities, functional decline of important organs (including kidney, liver, and brain), platelet hyperreactivity, hemodynamic instability, combinatory usage of multiple medications, and frailty (Ariza-Solé et al., 2014; Shanmugam et al., 2015; Schoenenberger et al., 2016; Arahata and Asakura, 2018; Martí et al., 2020). Herein, the incidence of BARC ≥2 type (excluding BARC four type) bleeding events in elderly patients was 3.94%, while the BleeMACS sub-study involving 3,376 ACS patients (aged ≥75 years) reported the incidence of severe bleeding after discharge to be 5.6% (Garay et al., 2018). Several other studies have shown a higher incidence of major bleeding events than that in our study (Sharma et al., 2017; Chen et al., 2019; Shimizu et al., 2019; Martí et al., 2020), which may be due to the inclusion of more lower bleeding risk subjects, such as stable CAD patients in our study.

Several bleeding risk scores have been proposed to predict the occurrence of bleeding complications (Costa et al., 2017; Pavasini et al., 2019). Previous research studies suggested that the CRUSADE score might be the most appropriate tool to predict major bleeding events. However, the score was proven to be suitable for predicting in-hospital bleeding, wherein Faustino A et al., reported less ability of the score to discriminate major bleeding events in NSTE-ACS octogenarians, thus indicating its limited application in elderly population (Subherwal et al., 2009; Faustino et al., 2014; Choi et al., 2018). The PRECISE-DAPT score is a simple tool with only five items from eight multicenter randomized clinical trials for weighing the risk of post-discharge bleeding to provide an effective tool for guiding the duration of DAPT (Choi et al., 2018; Costa et al., 2019a). PRECISE-DAPT series had a fewer involvement of older patients with their mean age ranging 60–65 years, while patients (≥75 years) accounted for only ∼25% (Choi et al., 2018; Guerrero et al., 2018). Since advanced age may push the PRECISE-DAPT score toward right, it is necessary to investigate whether the PRECISE-DAPT scoring system is still suitable for assessing the bleeding risk in elderly CAD patients, amidst the criteria for determining an optimal cutoff value for this score remain unanswered. There are limited studies on the performance of the PRECISE-DAPT score in older patients. Our results demonstrated that the PRECISE-DAPT score has the potential to predict BARC ≥2 type bleeding events in elderly CAD patients who received DAPT following PCI, despite its limited predictive ability upon applying the recommended cutoff value of 25. Previous study further suggested that a cutoff value of the PRECISE-DAPT score of ≥25 might be too low for elderly patients to be used in clinical practice (Costa et al., 2019b). In this regard, we stratified the PRECISE-DAPT score into tertiles (T1:≤23; T2:24 to 32; T3:≥33) and found that BARC ≥2 type bleeding events occurred more frequently in T3 versus T1 [odds ratio: 3.772; 95% confidence interval (1.229, 11.578); *p* = 0.02]. Interestingly, the comparison between the AUCs under the ROC curves of the current recommended cutoff value of 25 and the newly proposed 33 *via* the Delong test further indicated significant improvement in the ability of latter to predict the one-year BARC ≥2 type bleeding events compared to the former. The IFFANIAM study confirmed that the quartiles of PRECISE-DAPT values, rather than the recommended 25 value, showed good performance in assessing bleeding risk in elderly STEMI patients (Guerrero et al., 2018). Therefore, further studies should be conducted on the performance of the PRECISE-DAPT score to evaluate the bleeding complications in the elderly patients.

Severe bleeding complications may result in an increased mortality, cessation of antiplatelet drugs, and potential ischemic events, especially in the elderly patients (Lopes et al., 2012; Ndrepepa et al., 2014; Palmerini et al., 2017b). Numerous data have indicated that Asians displayed a higher bleeding risk than the western natives (Levine et al., 2014). Therefore, it is important for us to identify risk factors of bleeding and perform accurate risk stratification. Our regression analysis confirmed that the PRECISE-DAPT score system was efficient for predicting BARC ≥2 type bleeding events in elderly patients with an optimized cutoff score. In addition, it was further observed in this study that the history of hemorrhagic stroke, peptic ulcer, and MI were the main risk factors to predict BARC ≥2 type bleeding complications in CAD patients (≥75 years) and those undergoing one-year DAPT following PCI. As elderly patients with a history of bleeding or MI had a higher risk of BARC ≥2 type bleeding, it is therefore important to tailor the DAPT regimen for patients (≥75 years) undergoing PCI. The usage of PPI was supposed to have a protective role to reduce the gastrointestinal bleeding risk in CAD patients who received antiplatelet treatment (Khan et al., 2019); however, no association was found between the use of PPI and the reduction of BARC ≥2 type bleeding events in elderly patients in our study. The reasons may be due to the irregular usage of PPIs in the clinics and the limited number of patients enrolled in our study. Therefore, more studies are needed to investigate the correlation between PPI use and the reduction of bleeding risk so as to guide the rational for using PPI in clinical practice.

Several limitations in our study should be considered. First, this is a retrospective study with a relatively small sample size and a lower incidence of bleeding complications in comparison with other studies. Second, bleeding events were reported by patients, which may lead to some inaccuracy and bias. Third, although we analyzed a number of possible risk factors, some other predisposing factors such as frailty, malignant tumor, and revascularization procedure were not assessed in our study.

In conclusion, our study revealed that the current recommended PRECISE-DAPT score cutoff value of 25 might be useful in predicting BARC ≥2 type bleeding events. However, a higher cutoff value of 33 was more suitable in predicting BARC ≥2 type bleeding complications among elderly patients (≥75 years) who received DAPT following PCI. In addition, a history of hemorrhagic stroke, peptic ulcer, and MI provided additional information for the prediction of BARC ≥2 type bleeding events in elderly patients who underwent PCI and one-year DAPT.

## Data Availability

The raw data supporting the conclusions of this article will be made available by the authors, without undue reservation.
