# Supplementary material for: Incidence and Risk Factors for Antiplatelet Therapy–Related Bleeding Complications Among Elderly Patients After Coronary Stenting: A Multicenter Retrospective Observation
Source: Front Pharmacol. 2021 Jul 30;12:661619. doi: 10.3389/fphar.2021.661619 (PMC8362353; doi:10.3389/fphar.2021.661619)
Supplement: Supplementary file 1 [file Table1.docx]

Supplementary Table 1 Bleeding Academic Research Consortium Definition for Bleeding

| Type 0 |
| --- |
| No bleeding |
| Type 1 |
| Bleeding that is not actionable and does not cause the patient to seek unscheduled performance of studies, hospitalization, or treatment by a healthcare professional; |
| May include episodes leading to self-discontinuation of medical therapy by the patient without consulting a healthcare professional |
| Type 2 |
| Any overt, actionable sign of hemorrhage (eg, more bleeding than would be expected for a clinical circumstance, including bleeding found by imaging alone) that does not fit the criteria for type 3, 4, or 5 but does meet at least one of the following criteria: |
| requiring nonsurgical, medical intervention by a healthcare professional |
| leading to hospitalization or increased level of care |
| prompting evaluation |
| Type 3 |
| Type 3a |
| Overt bleeding plus hemoglobin drop of 3 to <5 g/dL* (provided hemoglobin drop is related to bleed) |
| Any transfusion with overt bleeding |
| Type 3b |
| Overt bleeding plus hemoglobin drop ≥5 g/dL* (provided hemoglobin drop is related to bleed) |
| Cardiac tamponade |
| Bleeding requiring surgical intervention for control (excluding dental/nasal/skin/hemorrhoid) |
| Bleeding requiring intravenous vasoactive agents |
| Type 3c |
| Intracranial hemorrhage (does not include microbleeds or hemorrhagic transformation, does include intraspinal) |
| Subcategories confirmed by autopsy or imaging or lumbar puncture |
| Intraocular bleed compromising vision |
| Type 4: CABG-related bleeding |
| Perioperative intracranial bleeding within 48 h |
| Reoperation after closure of sternotomy for the purpose of controlling bleeding |
| Transfusion of ≥5 U whole blood or packed red blood cells within a 48-h period |
| Chest tube output ≥2L within a 24-h period |
| Type 5: fatal bleeding |
| Type 5a |
| Probable fatal bleeding; no autopsy or imaging confirmation but clinically suspicious |
| Type 5b |
| Definite fatal bleeding; overt bleeding or autopsy or imaging confirmation |
